# A Novel Algorithm for the Determination of Walker Damage in Loaded Disc Springs

**DOI:** 10.3390/ma13071661

**Published:** 2020-04-03

**Authors:** Max Benedikt Geilen, Marcus Klein, Matthias Oechsner

**Affiliations:** Centre for Engineering Materials (MPA-IfW); Technical University of Darmstadt; Grafenstraße 2, 64283 Darmstadt, Germany; m.klein@mpa-ifw.tu-darmstadt.de (M.K.); oechsner@mpa-ifw.tu-darmstadt.de (M.O.)

**Keywords:** disc springs, fatigue, FEA, automation, real geometry, Abaqus Scripting

## Abstract

In this paper, a novel algorithm for the determination of Walker damage in loaded disc springs is presented. The algorithm takes a 3D-scan of a disc spring, measured residual stresses, material parameters, and spring loads as inputs. It outputs a distribution of Walker damage over the surface area of the input disc spring. As the algorithm allows a fully automated determination of the Walker damage, it can be used by disc spring manufacturers to reduce the working time spent on this task by specialized engineers significantly. Compared to spreadsheet applications using analytical formulas and finite element models using idealized geometry, this approach offers a superior description of the stress states in disc springs.

## 1. Introduction

The fatigue behavior of disc springs can be described by models ranging widely in complexity. Simple models like the one described in [1,2] can be implemented using spreadsheet applications, e.g., Microsoft Excel. As models become more sophisticated, the expense of building and evaluating the model rises. With the latest step in creating more complex models, the introduction of scanned geometries, superposed residual stresses, and the Walker damage parameter, the need for a novel algorithm for the determination of Walker damage in loaded disc springs has arisen.

The algorithm described in this paper is implemented in the Spring_stack Python module, which can also be used for other applications. For more information on the simulation of single disc springs without a 3D-scanned geometry or with multiple springs in one assembly, see [3,4].

The algorithm is the first published algorithm to build and evaluate a finite element model from a 3D-scanned geometry without user interaction. It allows the user to obtain an understanding of the influence of geometric deviations that are present in a batch of disc springs on the lifetime of individual disc springs under cyclic loading. This would be impossible without the algorithm because manually conducting a finite element simulation for each disc spring is prohibitively expensive.

Introductions to the mathematical description of disc springs and to the Walker damage parameter are given in Section 2. In Section 3, an algorithm used to describe Walker damage at the surface of a 3D-scanned disc spring is presented. Its implementation is described in Section 4. An example application is presented in Section 5. We recommend that readers without programming experience read Section 5 before Section 4.

## 2. Related Research

### 2.1. Mechanical Behavior of Disc Springs

The first mention of conical disc springs with a rectangular cross-section in the literature was Belleville’s patent in 1861 [5,6,7]. The first formulas to compute the characteristic of disc springs, as well as the stresses present in disc springs under load were published by Almen and Laszlo in 1936. These formulas are still in use in today’s standards [1,2], supplemented by a friction formulation published by Curti and Montanini [8]. There is a variety of other approaches for the analytical assessment of disc springs, each bringing its own advantages, such as improved accuracy [9,10,11,12,13,14,15,16,17,18,19], improved simplicity [20], applicability for different geometries [21,22,23,24,25,26,27,28], consideration of new material laws [29,30,31], offering a new concept for friction [32], or allowing the computation of resonance frequencies [33].

Analytical assessment of disc springs allows a direct understanding of the mechanical nature of disc springs and even the direct identification of links between geometric features and stresses. It also offers a sufficiently precise description of disc springs for applications like fatigue design according to EN 16983 and EN 16984 [1,2] at low computational costs. They can also be automated easily, which makes these approaches attractive for optimization algorithms like [34,35] and for analytical models of systems containing multiple components like [36,37].

Since the 1980s [38], disc springs have also been assessed using finite element analysis (FEA). While FEA is expensive in licensing, computational cost, and training, it allows models to be adapted to new, similar problems quickly. The first FEA models were used to exceed the accuracy of the analytical approaches available at that time [39,40]. FEA is still in use for the verification of new analytical models. FEA has extensively been used to describe residual stresses and changes in the characteristics created by plastic deformation and creep effects [41,42,43,44,45,46]. It has also been used to describe disc springs with complex geometries [47,48,49,50,51] or complex load cases [52], as well as for disc springs made from new materials [48,53,54,55,56,57] and to describe the behavior of assemblies containing disc springs [58,59,60].

Today, 2D models of disc springs solve quickly. An automated tool for the processing of 2D simulations of disc springs was implemented as early as 2000 [61]. FEA has been included in the curricula of most engineering programs. Therefore, graduates with basic FEA skills are available to companies. Disc springs have been numerically simulated by free finite element software [62]. These effects have led to the literature being split roughly in half between analytical formulas and FEA. The authors believe this distribution to roughly stay the same in the future because both approaches offer unique advantages and because of a legacy effect. The legacy effect is created by fatigue tests being evaluated using a certain method and the raw data like material laws and spring geometries not being documented. Furthermore, engineers are more experienced in the design of springs using analytical formulas, and this experience can only partially be used for the design of springs using FEA.

### 2.2. The Walker Damage Parameter

The Walker damage parameter [63] helps to compare the fatigue behavior of materials under different mean stresses. Compared with other approaches to the characterization of mean stress effects in steels [64,65,66], the Walker damage parameter shows a superior lifetime prediction [67]. In this paper, the stress-based approach is described. For the strain-based approach, see [68].

The Walker damage parameter $P_{\mathrm{Walker}}$ is computed from the maximum stress $\sigma_{\max}$, the stress amplitude $\sigma_{\mathrm{amp}}$, and the Walker exponent γ:

(1) $$P_{\mathrm{Walker}}=\sigma_{\max}^{1-\gamma }\sigma_{\mathrm{amp}}^{\gamma }$$

The Walker exponent γ is a material parameter that can be identified by fitting fatigue-curves with different mean stresses or R-ratios. A high Walker exponent implies a low sensitivity to mean stress effects, while a low Walker exponent implies a high sensitivity. If the Walker exponent γ is fixed to 0.5, the stress Walker approach is equivalent to the stress Smith–Watson–Topper [66] approach.

The Walker equation requires $\sigma_{\max}$ and $\sigma_{\mathrm{amp}}$ to be scalar values. At the surface of the disc spring, the stress state is two-dimensional; therefore, an equivalent stress must be computed. Here, the von Mises equivalent stress is used.

For proportional loads in Quadrant 1, the computation of the maximum stress $\sigma_{\max}$ and the stress amplitude $\sigma_{\mathrm{amp}}$ from the minimum and maximum principal stresses $\sigma_{I,\max}$, $\sigma_{\mathrm{II},\max}$, $\sigma_{I,\min}$, and $\sigma_{\mathrm{II},\min}$ is obvious.

(2) $$\sigma_{\min}=\sqrt{\frac{\sigma_{I,\min}^{2}+{\left(\sigma_{I,\min}-\sigma_{\mathrm{II},\min}\right)}^{2}+\sigma_{\mathrm{II},\min}^{2}}{2}}$$

(3) $$\sigma_{\max}=\sqrt{\frac{\sigma_{I,\max}^{2}+{\left(\sigma_{I,\max}-\sigma_{\mathrm{II},\max}\right)}^{2}+\sigma_{\mathrm{II},\max}^{2}}{2}}$$

(4) $$\sigma_{\mathrm{amp}}=0.5\cdot \left(\sigma_{\max}-\sigma_{\min}\right)$$

**Figure 1:**
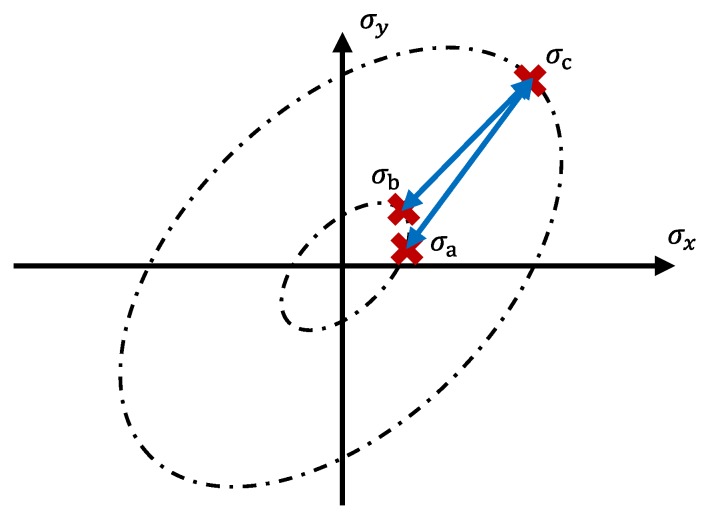
Planar stress with the von Mises equivalent (zero shear).

The stress state in disc springs however is non-proportional, especially in shot peened specimens. An example why the formulas given for the proportional case may give inconsistent results for the non-proportional case is depicted in Figure 1. Given the stress states $\sigma_{a}$, $\sigma_{b}$, and $\sigma_{c}$ in Quadrant 1, the stress amplitude between $\sigma_{a}$ and $\sigma_{c}$ is equal to the stress amplitude between $\sigma_{b}$ and $\sigma_{c}$ if the von Mises equivalent stress in $\sigma_{a}$ is equal to the von Mises equivalent stress in $\sigma_{b}$. The same applies for mean stresses. In reality, the damage inflicted by cycling between $\sigma_{b}$ and $\sigma_{c}$ is smaller than the damage inflicted by cycling between $\sigma_{a}$ and $\sigma_{c}$.

To avoid the described misrepresentation of damage inflicted, the modified Manson–McKnight method [69] is used. Instead of computing two scalar stress states $\sigma_{\max}$ and $\sigma_{\min}$ and deducing a scalar amplitude and a scalar mean stress, a two-dimensional stress amplitude and a two-dimensional mean stress are deduced from a two-dimensional maximum and minimum stress state:

(5) $$\sigma_{I,\mathrm{amp}}=0.5\cdot \left(\sigma_{I,\max}-\sigma_{I,\min}\right)$$

(6) $$\sigma_{\mathrm{II},\mathrm{amp}}=0.5\cdot \left(\sigma_{\mathrm{II},\max}-\sigma_{\mathrm{II},\min}\right)$$

(7) $$\sigma_{I,\mathrm{mean}}=0.5\cdot \left(\sigma_{I,\max}+\sigma_{I,\min}\right)$$

(8) $$\sigma_{\mathrm{II},\mathrm{mean}}=0.5\cdot \left(\sigma_{\mathrm{II},\max}+\sigma_{\mathrm{II},\min}\right)$$

These pairs of stress components are converted into von Mises equivalent stresses:

(9) $$\sigma_{\mathrm{amp}}=\sqrt{\frac{\sigma_{I,\mathrm{amp}}^{2}+{\left(\sigma_{I,\mathrm{amp}}-\sigma_{\mathrm{II},\mathrm{amp}}\right)}^{2}+\sigma_{\mathrm{II},\mathrm{amp}}^{2}}{2}}$$

(10) $$\sigma_{\mathrm{mean}}=\frac{\sigma_{I,\mathrm{mean}}-\sigma_{\mathrm{III},\mathrm{mean}}}{\sigma_{I,\mathrm{mean}}+\sigma_{\mathrm{III},\mathrm{mean}}}\cdot \sqrt{\frac{\sigma_{I,\mathrm{mean}}^{2}+{\left(\sigma_{I,\mathrm{mean}}-\sigma_{\mathrm{II},\mathrm{mean}}\right)}^{2}+\sigma_{\mathrm{II},\mathrm{mean}}^{2}}{2}}$$

The maximum stress $\sigma_{\max}$ is defined with a lower bound to avoid complex numbers as Walker damage parameters.

(11) $$\sigma_{\max}=\max\left(\sigma_{\mathrm{mean}}+\sigma_{\mathrm{amp}},0\right)$$

## 3. The Algorithm

### 3.1. General Concept

The algorithm starts with a surface mesh (given as an .STL file) of a disc spring placed randomly in space. After aligning the disc spring approximately symmetrically around the y-axis, it imports the geometry information into a finite element application. In our implementation of the algorithm, the finite element application was used. Through the Abaqus Scripting interface [70,71], it builds a model around the geometry data, using additional inputs like loads and friction coefficients provided by the user. It generates an output database file using the Abaqus/Standard Solver. Utilizing Abaqus/Viewer functionalities via the Scripting interface, a field of residual stresses is generated from input data. Aggregated minimum and maximum stress fields are computed by superposing the computed load stress field and the residual stress field. A Walker damage parameter field is computed from the minimum and maximum stress fields. The Walker damage parameter at the surface of the disc spring is computed and exported as tabular data.

### 3.2. Architecture

**Figure 2:**
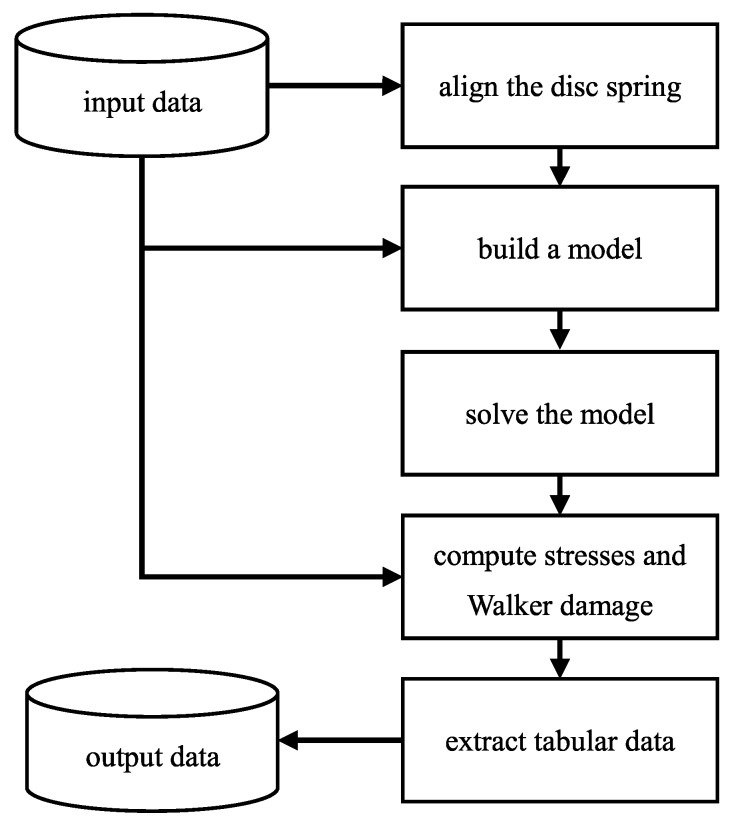
Simplified flowchart of the algorithm.

The software architecture used is called a pipeline in programming terms and resembles a production line: an instance of the class Spring_stack is passed through a series of different processing stations. The processing stations gradually transform the instance from input data to a solved finite element model with post-processed output fields and from there to easily readable tabular data and a graphical representation thereof. In programming terms, these processing stations are called methods. The flow of data is depicted in Figure 2.

### 3.3. User-provided Inputs

To conduct the inquiry described above, the algorithm requires several inputs. These inputs are provided by the user. They are comprised of an .STL file containing the triangle surface mesh of a scanned disc spring and one .JSON file for the description of the simulated physical situation and the numerical configuration of the simulation, respectively.

The .JSON file describing the simulated physical situation contains an array of parts defining the geometry, the Walker exponent γ, Young’s modulus, the Poisson number, and a volumetric mass density (for numerical stability) for each. The geometry of scanned disc springs is given by means of a link to an .STL file. Plasticity may be defined for disc springs in the present implementation. However, this is not in the scope of this paper. Furthermore, the friction coefficients between springs stacked in parallel and springs stacked in series, as well as between springs and the plates and the pillar are committed. The axial load is committed as an array of paths, giving the displacements of one of the plates at the end of each time step. Measured residual stresses in the radial and tangential direction are committed for two points with different coordinates. These two points should be selected carefully by the user and ideally describe a linear model representing more measurements. Furthermore, a target value for the number of triangles of the coarsened triangle surface mesh is committed.

The .JSON file describing the numeric inputs contains several naming definitions, element types, solver options, contact and friction formulations, computing resource allowances, and flags determining whether stresses etc. are to be written to input files. Different modeling conventions for plates and pillars may be used in the current implementation. However, this is outside the scope of this paper.

## 4. Implementation

### 4.1. Aligning the Disc Spring

In order to build a model around a part, the alignment of the part in space must be known. For example, to apply axial loads, the axial direction of the loaded part must be known. Because it makes the rest of the algorithm much easier, the input disc spring is preprocessed so it always has the same orientation. By convention, this position is in the point of origin, closely axially symmetric to the *y*-axis.

In the alignment process, the mesh $M_{\mathrm{start}}$ is processed as a set of vertices $V_{\mathrm{start}}=\left\{v_{1},\cdots ,v_{v}\right\}$ and a set of triangles $e=\{e_{1},\cdots ,e_{e}\}$ connecting these vertices. The vertices $v_{i,\mathrm{start}}=\left(x_{i,\mathrm{start}},y_{i,\mathrm{start}},z_{i,\mathrm{start}}\right)$ are defined in a Cartesian coordinate system (x,y,z). The point set is aligned by a translation $t\left(t_{x},t_{y},t_{z},V\right)$ along and a rotation $A\left(\theta_{x},\theta_{y},\theta_{z}\right)$ around the principal axes.

(12) $$V_{\mathrm{aligned}}\left(A,t,V_{\mathrm{start}}\right)=A\left(\theta_{x},\theta_{y},\theta_{z}\right)\cdot t\left(t_{x},t_{y},t_{z},V_{\mathrm{start}}\right)$$

The edges are defined as connectors between vertices; therefore, a geometric transformation of any mesh M is fully described by a geometric transformation of its vertices V. The resulting mesh $M_{\mathrm{aligned}}$ is defined by the vertices $V_{\mathrm{aligned}}$ and the edges e.

A feasible combination of translation t and rotation A is found by solving an optimization problem.

(13) $$\min_{\theta_{x,y,z},t_{x,y,z}}f\left(V_{\mathrm{aligned}}\left(A,t,V_{\mathrm{start}}\right)\right)$$

To compute the objective function *f*, the transformed point cloud $V_{\mathrm{aligned}}$ is projected into the xy-plane, producing $V_{xy}=\left\{v_{xy,1},\cdots ,v_{xy,v}\right\}$ by rotation around the *y*-axis. $V_{xy}$ is computed using the coordinates of $V_{\mathrm{aligned}}$:

(14) $$v_{xy,i}=\left(\begin{array}{c} \sqrt{x_{i,\mathrm{aligned}}^{2}+y_{i,\mathrm{aligned}}^{2}}\\ z_{i,\mathrm{aligned}} \end{array}\right)$$

The objective function *f* is defined as the area *A* of the convex hull of $V_{xy}$.

(15) $$f=A\left(Conv\left(V_{xy}\right)\right)$$

The convex hull is computed using the Quickhull algorithm [72,73]. The objective function *f* is invariant to rotations around the *y*-axis. Therefore, $\theta_{y}$ is fixed to zero. To further simplify the optimization problem, it is assumed that the centroid of the aligned mesh $c\left(M_{\mathrm{aligned}}\right)$ lies in the point of origin. Although this assumption is applicable only for perfectly symmetric spring geometries, we assumed that for springs possessing minor deviations regarding their symmetry, the assumption holds true, too.

Based on this assumption, t is defined as translating the volumetric centroid *c* of the mesh $M_{\mathrm{start}}$ into the point of origin.

(16) $$c\left(M_{\mathrm{translated}}\right)=\left(0,0,0\right)$$

The computational cost for finding t is low because the volumetric centroid of any closed mesh M can be computed inexpensively.

In the following rotation A, the mesh $M_{\mathrm{translated}}$ is only rotated around the principal axes. As the volumetric centroid lies on all three principal axes, it is invariant under said rotation A.

(17) $$c\left(M_{\mathrm{aligned}}\right)=c\left(M_{\mathrm{translated}}\right)=\left(0,0,0\right)$$

By fixing the translation additionally to the rotation around the *y*-axis, the dimensionality of the optimization problem is reduced from six to two.

(18) $$\min_{\theta_{x,z}}f\left(V_{\mathrm{aligned}}\left(A,t,V_{\mathrm{start}}\right)\right)$$

The parameters $\theta_{x}$ and $\theta_{z}$ are calculated using the L-BFGS-B method [73,74,75].

The resulting mesh $M_{\mathrm{aligned}}$ may be aligned upside down. This being the case, it can simply be rotated by 180 degrees around the *x*-axis. To further ease the following steps, it is translated along the *y*-axis, so its lowest point is in the xz-plane.

(19) $$\min\left(y_{i,\mathrm{postprocessed}}\right)=0$$

The postprocessed, aligned mesh $M_{\mathrm{postprocessed}}$ is exported as an Abaqus input file (.inp).

### 4.2. Building the Finite Element Model

**Figure 3:**
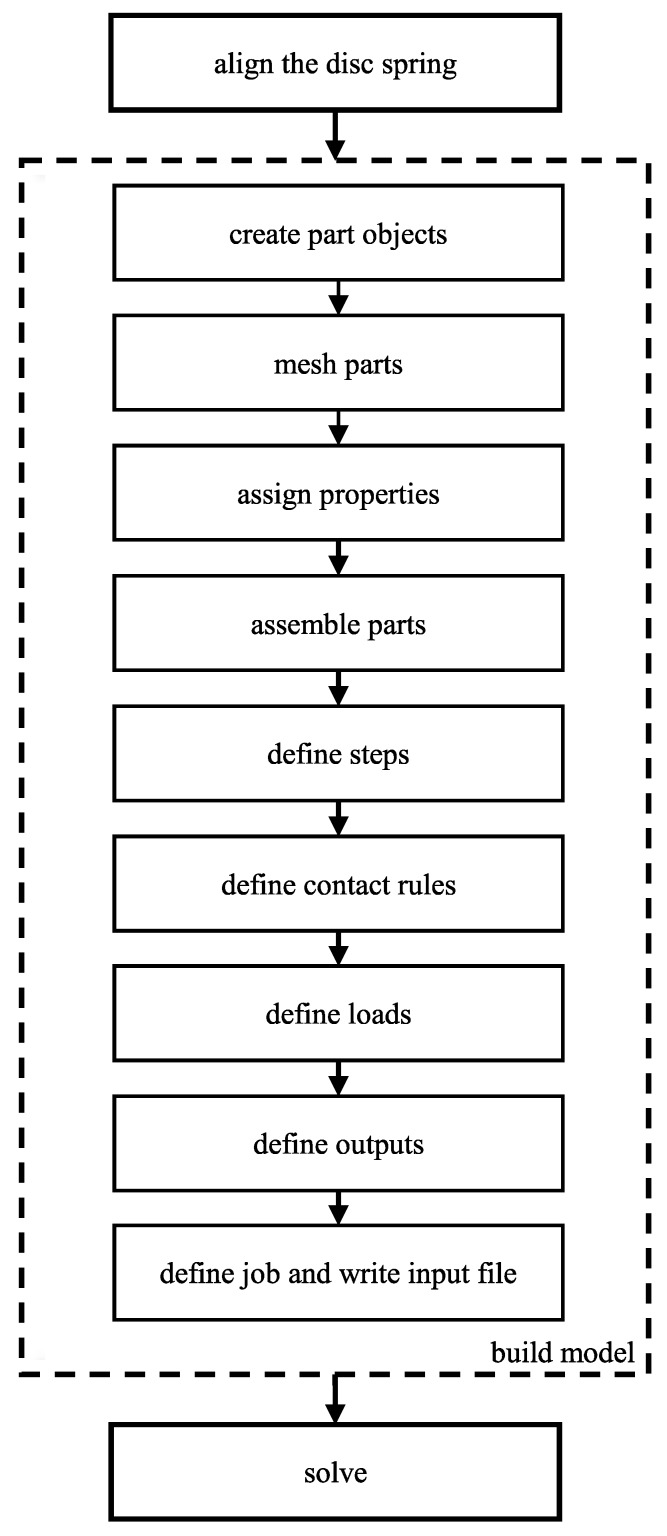
Flowchart of the sub-pipeline ’build model’.

The creation of the model is structured as a pipeline inside the pipeline depicted in Figure 2. The sub-pipeline is depicted in Figure 3. The allotment of tasks to processing stations (methods) along the pipeline is based on the allotment of functionalities to modules in the Abaqus/CAE environment. Each of the processing stations is customized according to input data. The following paragraphs describe the individual processing stations.

For the simulation of a single disc spring, four components are required: the disc spring itself, a guide pin, and two flat plates. The geometry of the disc spring is read from the input file generated from the 3D-scan data. The guide pin is defined as an idealized cylinder with the same height as the disc spring, and the plates are defined as idealized planes.

The plate and the guide pin part instances are defined as analytic rigid surfaces and therefore do not need to be meshed. The part instance representing the disc spring is imported as a triangle surface mesh and is converted into a volume tetrahedron mesh by the free mesher implemented in Abaqus/CAE. Mesh size cannot be controlled actively, but only by changing the mesh size of the imported surface mesh. The surface mesh size is adjusted by exporting very fine surface meshes from the 3D-scanning software GOM Scan and increasing the mesh size using the quadric edge collapse decimation algorithm [76]. In this application, quadric edge collapse is especially suitable because it reduces the mesh size at the flat surfaces, where the mesh may be more coarse, and keeps it nearly constant at the edges, where a fine mesh is needed.

In the approach described in this paper, the finite element analysis utilizes a purely elastic material law. Residual stresses are incorporated by superposition of the measured stresses committed by the user. This is implemented by building two axisymmetric field outputs, one for $\sigma_{\mathrm{residual},\tan}$ and one for $\sigma_{\mathrm{residual},\mathrm{rad}}$, in Abaqus/CAE. These output fields will later be used for the computation of $\sigma_{I,\min}$, $\sigma_{I,\max}$, $\sigma_{\mathrm{II},\min}$, and $\sigma_{\mathrm{II},\max}$ according to Equations (24) to (27).

To introduce inertia and improve convergence, a volumetric mass density is defined for the disc spring. Because the spring was aligned beforehand, the assembly is straightforward. All four parts are joined in an assembly with a single coordinate system. The lower plate, the disc spring, and the guide pin are already in place. The upper plate is positioned in its place by a translation along the *y*-axis.

The lower plate and the guide pin are fixed in space, and loads are applied to the upper plate. To improve convergence, a small gravitational load is defined. No other loads are applied directly to the disc. Forces are transmitted via contacts. Surface to surface contacts are defined between the disc spring and each of the other part instances.

The Abaqus Scripting interface includes an option to customize output requests. This option is used in the algorithm to request stress and coordinate outputs. However, coordinates cannot be requested at integration points by means of the Scripting interface. Coordinate data in the integration points are necessary to compute residual stresses in the integration points.

A job instance is created to generate an input file. The input file is manipulated to create an output request for coordinate information in the integration points, and a second job instance pointing to the new input file is created. This way, a job is created that is identical to the first job except that it includes an output request for coordinate information in the integration points. The second job is converted into a system of partial differential equations and solved using Abaqus/Standard.

### 4.3. Computing Stresses and Walker Damage Parameters

**Figure 4:**
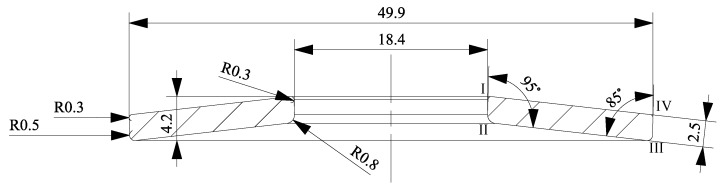
Idealized geometry of the spring under investigation.

After solving the system of partial differential equations for the displacements of the nodes in the model, Abaqus/Standard computes stresses and coordinates in the integration points at different points in step-time, which refer to the minimum and the maximum load. In this section, the computation of Walker damage parameters at the surface of the disc spring between Edge II and Edge III (see Figure 4) is described. Here, we may assume a plane stress state with a dominating tension component:

(20) $$\sigma_{\mathrm{load},\mathrm{II},\min}=0\vee \sigma_{\mathrm{load},\mathrm{III},\min}=0$$

(21) $$\sigma_{\mathrm{load},\mathrm{II},\max}=0\vee \sigma_{\mathrm{load},\mathrm{III},\max}=0$$

For integration points close to the surface, this is approximately true. The assumption becomes more realistic with a finer mesh and is true with an infinitesimal mesh size. The minimal and maximal stress components $\sigma_{\mathrm{load},\mathrm{II},\min}^{\prime }$ and $\sigma_{\mathrm{load},\mathrm{II},\max}^{\prime }$ are redefined:

(22) $$\sigma_{\mathrm{load},\mathrm{II},\min}^{\prime }=\sigma_{\mathrm{load},\mathrm{II},\min}+\sigma_{\mathrm{load},\mathrm{III},\min}$$

(23) $$\sigma_{\mathrm{load},\mathrm{II},\max}^{\prime }=\sigma_{\mathrm{load},\mathrm{II},\max}+\sigma_{\mathrm{load},\mathrm{III},\max}$$

In the surface of disc springs, $\sigma_{\mathrm{load},I}$ points in the tangential direction and $\sigma_{\mathrm{load},\mathrm{II}}^{\prime }$ points in the radial direction. Residual stresses are measured in the tangential direction ($\sigma_{\mathrm{residual},\tan}$) and in the radial direction ($\sigma_{\mathrm{residual},\mathrm{rad}}$). The input stresses for the computation of the Walker damage parameters are defined as:

(24) $$\sigma_{I,\max}=\sigma_{\mathrm{load},I,\max}+\sigma_{\mathrm{residual},\tan}$$

(25) $$\sigma_{\mathrm{II},\max}=\sigma_{\mathrm{load},\mathrm{II},\max}^{\prime }+\sigma_{\mathrm{residual},\mathrm{rad}}$$

(26) $$\sigma_{I,\min}=\sigma_{\mathrm{load},I,\min}+\sigma_{\mathrm{residual},\tan}$$

(27) $$\sigma_{\mathrm{II},\min}=\sigma_{\mathrm{load},\mathrm{II},\min}^{\prime }+\sigma_{\mathrm{residual},\mathrm{rad}}$$

Walker damage parameters are computed according to Equations (1) to (11). For each surface triangle, a surface area $A_{i}$ and a Walker damage parameter $P_{\mathrm{Walker},i}$ are computed. The surface Walker damage parameter is computed by averaging the Walker damage parameters of the neighboring nodes. These data pairs are saved as the set *S*.

### 4.4. Extracting Tabular Data

The set *S* is too rich in information to be captured holistically without extraction and/or condensation. This is done by transforming the information into tabular data. Therefore, it is condensed into human readable tabular data. Each element of the set is defined as a Dirac delta function:

(28) $$f_{i}\left(P_{\mathrm{Walker}}\right)=A_{i}\cdot \delta \left(P_{\mathrm{Walker}}-P_{\mathrm{Walker},i}\right)$$

The accumulated surface area $A_{\mathrm{acc}}$ assigned to a given Walker damage parameter is computed as:

(29) $$A_{\mathrm{acc}}\left(P_{\mathrm{Walker}}\right)=\int_{P_{\mathrm{Walker}}}^{\infty }\sum_{i}f_{i}\left(P_{\mathrm{Walker}}\right)dP_{\mathrm{Walker}}$$

It describes the size of the surface area of the disc spring with a Walker damage equal to or greater than the given $P_{\mathrm{Walker}}$. A graphical representation [77] of the accumulated surface area is created.

## 5. Example Application

**Figure 5:**
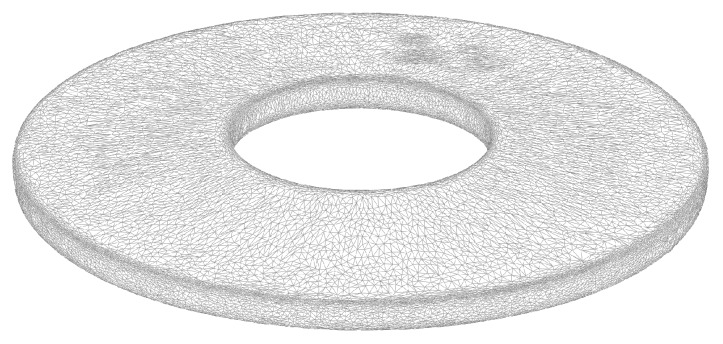
3D-scanned, coarsened surface mesh of the disc spring under investigation.

In this section, an example application of the algorithm presented in Section 3 is given. A single disc spring was modelled. The surface mesh of the disc spring under investigation is presented in Figure 5. The mesh was obtained using the commercially available 3D-scanning device GOM ATOS and the software GOM Scan. The number of surface triangles was already reduced for the displayed mesh, from 253,904 to 50,152. Convergence studies with different loads showed this mesh to be a good compromise between computational cost (about seven hours of CPU time on a i7-9800X and 16 GB of RAM vs. about 110 h of CPU time and 115 GB of RAM for the 253,904 surface triangle model) and accuracy (no significant bias of the Walker damage-surface area plot) [4].

**Figure 6:**
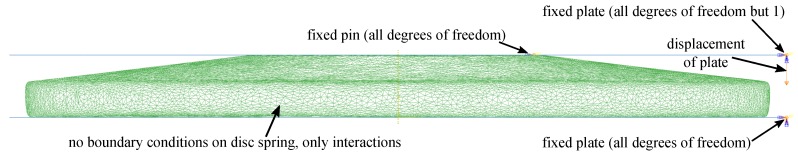
Boundary conditions defined for the finite element model.

Additionally, an idealized geometry derived from the scanned data is presented in Figure 4. Edges I to IV are labelled for the reader’s orientation. A major difference between both geometries is that the surfaces between Edges I and IV, as well as II and III of the idealized geometry are straight, while those of the 3D-scanned disc spring are curved. This is also visible in Figure 6. Of course, the 3D-scanned geometry also was not perfectly symmetric.

**Figure 7:**
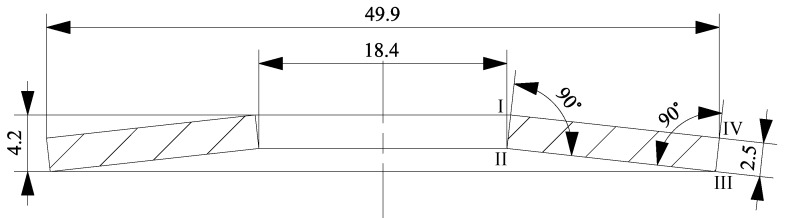
Geometry of the spring under investigation according to EN 16983 [1].

In Figure 7, the geometry according to the standard [1] is presented. It differs from the idealized geometry in having sharp edges and all angles between faces being 90°. This simplified geometry is usually utilized for the analysis of disc springs, regardless of whether analytical formulas or finite element models are used.

The triangle surface mesh was aligned and imported into Abaqus/CAE. Afterwards, it was converted into a tetrahedron volume mesh using the generateMesh method included in Abaqus Scripting [71]. The resulting linear tetrahedron C3D4 mesh was converted into a quadratic C3D10 mesh. Modified quadratic tetrahedrons C3D10M offer improved contact behaviour [78]; however, due to poor mesh quality, C3D10 tetrahedrons performed better in our experience. The elements were assigned a Young’s modulus of 206,000 MPa, a Poisson ratio of 0.3, and a volumetric mass density of 8.05 g/mm${}^{3}$.

The volumetrically meshed disc spring was incorporated into an assembly. The load cycle was implemented in four steps. To obtain good convergence behavior, the steps were defined as dynamic steps. The implicit solver Abaqus/Standard was used. The purpose of Step 1 was to apply the lower load. Step 2 was to make sure there was no dynamic influence on the computed stresses. Step 3 was to apply the higher load. Step 4 was, again, implemented to eliminate dynamic influences. Step times for Steps 1 to 4 were 10 s, 1 s, 10 s. and 1 s. The boundary conditions were applied to the upper plate as displacements in the axial direction at a reference point; see Figure 6. The prescribed displacements were 0.425 mm for Steps 1 and 2 and 1.19 mm for Steps 3 and 4. All other degrees of freedom of the reference points were fixed to zero. For the lower plate, all degrees of freedom were fixed to zero.

All contact formulations used in this model were defined as surface-to-surface contacts with a finite sliding penalty formulation and a friction coefficient of 0.01.

**Figure 8:**
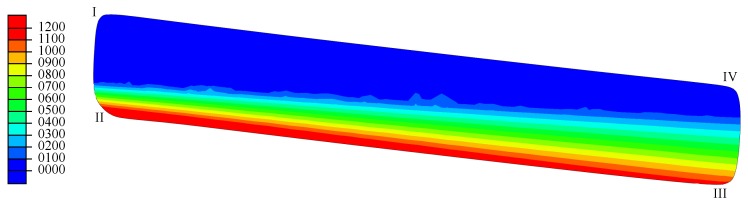
Computed compressive (deep blue, without detailed scale) and computed highest tensile (colored scale) load stresses in the fully loaded cross-section in MPa.

A job was created and committed to the solver Abaqus/Standard. The resulting output database was loaded. The resulting highest tensile load stresses in a cross-section are displayed in Figure 8. The upper half of the disc spring was loaded compressively. This is why disc springs in general break between Edges II and III.

**Figure 9:**
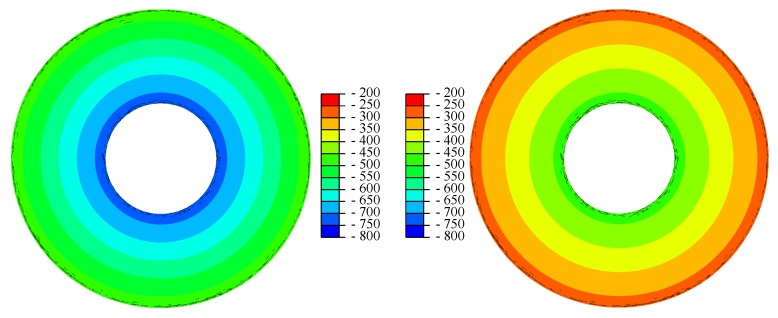
Residual stresses $\sigma_{\mathrm{residual},\tan}$ and $\sigma_{\mathrm{residual},\mathrm{rad}}$ in the tangential (**left**) and radial (**right**) direction in MPa.

Two field outputs representing residual stresses in the tangential and radial direction were generated based on user input and the initial coordinates of the integration points (which were requested as outputs earlier). Since the measured residual stresses for the spring under investigation are confidential, the used inputs values were not the result of a measurement. They were however realistic for disc springs. The residual stresses between Edges II and III were approximated by a linear function, ignoring non-symmetric effects. The computed residual stresses were obviously wrong anywhere else. This does not matter here because the Walker damage parameter is a measure used to predict fracture. Fracture is initiated by cracks, which normally initiate from the surface between Edges II and III [79]. Cracks originating from between Edges I and IV are usually caused by too low preloading forces. The bias in the Walker damage parameter introduced outside the surface between Edges II and III is non-conservative. Therefore a false positive for fracture in this area can be ruled out. The computed output fields between Edges II and III are displayed in Figure 9. The significantly higher residual stresses in the tangential direction compared to the radial direction are normal in disc springs because disc springs are overloaded in production to prevent plastic deformation in use, to increase lifetime, and to decrease creep effects [80,81,82,83,84,85].

The output fields resulting from the finite element simulation describing stresses after Step 2 and the output fields describing the residual stresses were added up to compute output fields describing $\sigma_{I,\max}$ and $\sigma_{\mathrm{II},\max}$. The calculation followed Equations (24) and (25). Field outputs describing $\sigma_{I,\min}$ and $\sigma_{\mathrm{II},\min}$ were computed following Equations (26) and (27).

**Figure 10:**
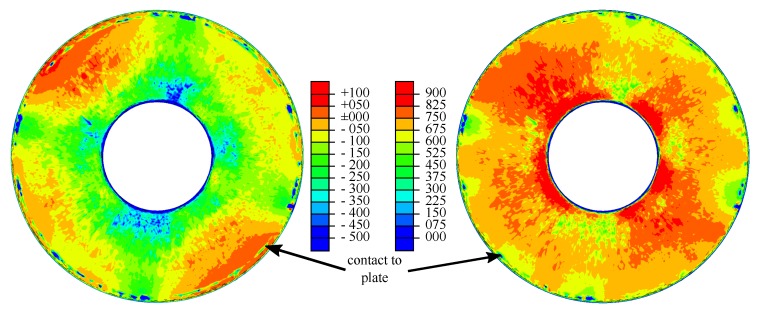
Minimum and maximum equivalent von Mises stress $\sigma_{\min}$ (**left**) and $\sigma_{\max}$ (**right**) in MPa.

Based on these, field outputs describing the minimum and maximum equivalent stress, $\sigma_{\min}$ and $\sigma_{\max}$, were computed according to Equations (2) and (3); see Figure 10. Especially in the minimum equivalent von Mises stress visualization, the contact line between the disc spring and the lower plate can be identified by a circle of locations with high compressive stresses.

**Figure 11:**
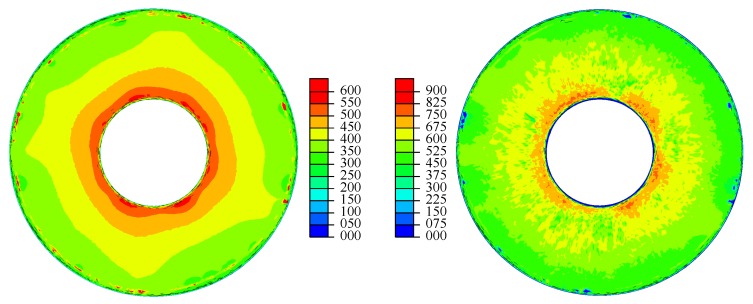
Stress amplitude $\sigma_{\mathrm{amp}}$ (**left**) and Walker damage $P_{\mathrm{Walker}}$ (**right**) in MPa.

These output fields were used to compute the output fields representing the stress amplitude $\sigma_{\mathrm{amp}}$ according to Equation (4) and finally the Walker damage parameter $P_{\mathrm{Walker}}$ according to Equation (1); see Figure 11. The Walker exponent γ=0.5 was used, which makes the Walker damage parameter equivalent to the Smith–Watson–Topper damage parameter. Compared to the other fields, the stress amplitude field was very smooth. The reason for this is that the elastic deformation of the spring partially compensated for the small asymmetries that were present. For higher load increments, the additional elastic stresses were therefore distributed more homogeneously.

**Figure 12:**
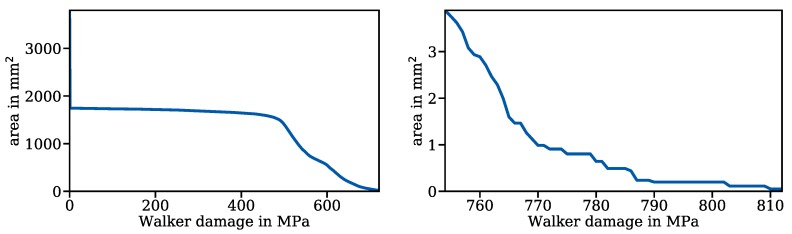
Accumulated surface area of the disc spring over Walker damage.

The algorithm created a list of all surface triangles and computed an average Walker damage parameter $P_{\mathrm{Walker},i}$, as well as a surface area $A_{i}$ for each triangle. The graph of the accumulated surface area over the Walker damage parameter was created; see Figure 12. From this particular graph, the user can for example extract the surface area where the Walker damage parameter is over 750 MPa, which is 4.9 mm${}^{2}$. About half of the surface had a Walker damage parameter of zero because stresses there were purely compressive; see Figure 12. This surface corresponds to the dark blue parts of the surface in Figure 8. As can be seen on the graph on the right, the function starts to jump at high stresses. This is because Walker damage parameters were averaged over surface triangles. The part of the graph at very high stresses exists purely because of numerical singularities and therefore does not correspond to the fatigue behaviour of the disc spring.

**Figure 13:**
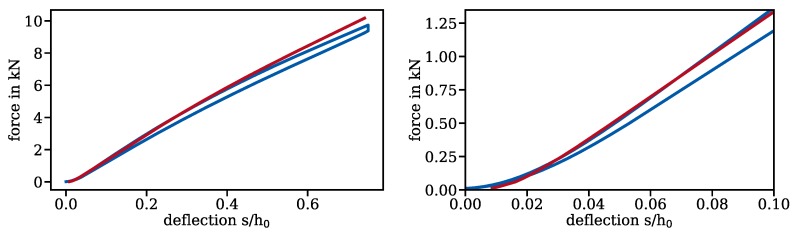
Comparison of characteristics obtained from numerical simulation and from the experiment.

The Walker damage parameter is not directly accessible through experiments. To evaluate the quality of the finite element model, a characteristic derived from a similar model (only boundary conditions were changed) was compared to a characteristic obtained in an experiment; see Figure 13. As is customary for disc springs, the deflection was normalized over the deflection at which the disc spring lies flat on the ground. They agree well; especially, the correct representation of the progressive behaviour at the very start of the experiment has only been achieved by models directly implementing 3D-scanned geometry. To our knowledge, all published models directly implementing 3D-scanned geometry have been created using the algorithm presented in Section 3. The numerical characteristic is somewhat stiffer than the experimental one. This may be due to a misrepresentation of Young’s modulus, which was set to the normative default of 206,000 MPa; however, tensile tests on specimens from the same batch of material and a similar heat treatment did not show a sufficient deviation in Young’s modulus to use a lower value.

## 6. Summary

A novel algorithm as implemented in the Spring_stack module was presented in this paper. The algorithm receives geometry data, residual stresses, material parameters, load cases, and further inputs and builds an FE model based on these. It evaluates the FE model after solving with respect to the Walker damage inflicted locally using a Manson–McKnight approach. In a post-processing step, the accumulated surface area as a function of the Walker damage parameter is computed. An example application of the algorithm was presented.
